# Loss of the mammalian G-protein coupled receptor, G2A, modulates severity of invasive pulmonary aspergillosis

**DOI:** 10.3389/fimmu.2023.1173544

**Published:** 2023-06-26

**Authors:** Breanne N. Steffan, Dante Calise, Sung Chul Park, Mengyao Niu, Jun Yang, Bruce D. Hammock, MaryJane Jones, Chad Steele, Nancy P. Keller

**Affiliations:** ^1^ Department of Medical Microbiology and Immunology, University of Wisconsin-Madison, Madison, WI, United States; ^2^ Department of Entomology, University of California-Davis, Davis, CA, United States; ^3^ Department of Microbiology and Immunology, School of Medicine, Tulane University, New Orleans, LA, United States; ^4^ Department of Plant Pathology, University of Wisconsin-Madison, Madison, WI, United States

**Keywords:** *Aspergillus fumigatus*, GPR132, G2A, oxylipin, neutrophil, lung, aspergillosis, hydroxyoctadecadienoic acid

## Abstract

**Background:**

*Aspergillus fumigatus* is a well-known opportunistic pathogen that causes a range of diseases including the often-fatal disease, invasive pulmonary aspergillosis (IPA), in immunocompromised populations. The severity of IPA is dependent on both host- and pathogen-derived signaling molecules that mediate host immunity and fungal growth. Oxylipins are bioactive oxygenated fatty acids known to influence host immune response and *Aspergillus* developmental programs. *Aspergillus* synthesizes 8-HODE and 5,8-diHODE that have structural similarities to 9-HODE and 13-HODE, which are known ligands of the host G-protein-coupled receptor G2A (GPR132).

**Materials and methods:**

Oxylipins were extracted from infected lung tissue to assess fungal oxylipin production and the Pathhunter β-arrestin assay was used to assess agonist and antagonist activity by fungal oxylipins on G2A. An immunocompetent model of *A. fumigatus* infection was used to assess changes in survival and immune responses for G2A-/- mice.

**Results:**

Here we report that *Aspergillus* oxylipins are produced in lung tissue of infected mice and *in vitro* ligand assays suggest 8-HODE is a G2A agonist and 5,8-diHODE is a partial antagonist. To address the hypothesis that G2A could be involved in the progression of IPA, we assessed the response of G2A-/- mice to *A. fumigatus* infection. G2A-/- mice showed a survival advantage over wild-type mice; this was accompanied by increased recruitment of G2A-/- neutrophils and increased levels of inflammatory markers in *A. fumigatus*-infected lungs.

**Conclusions:**

We conclude that G2A suppresses host inflammatory responses to *Aspergillus fumigatus* although it remains unclear if fungal oxylipins are involved in G2A activities.

## Introduction

Invasive pulmonary aspergillosis (IPA), the most common filamentous fungal infection in patients with reduced innate immunity, impacts over 250,000 patients each year with up to 90% mortality (1–3). The leading infectious agent that causes IPA is *Aspergillus fumigatus*, an opportunistic and ubiquitous fungus with conidia small enough to penetrate deep into the lower respiratory tract when inhaled (4, 5). IPA can be found as a comorbidity in immunocompromised patients, including those with HIV/AIDs, cancer, and those on immunosuppressants (5). Most recently *A. fumigatus* infections have been associated with SARS-Cov2 leading to the severe disease known as COVID-19-associated pulmonary aspergillosis (CAPA) (6). Early immunological responses to *A. fumigatus*, including the release of pro-inflammatory signals, innate immune cell activation, and cell trafficking, are essential in the prevention of invasive disease. A critical and initial step in host response is recognition of the fungus through multiple host receptors.

Receptors have a well-characterized role in *A. fumigatus* infections including in the initial stages of recognition (7). Dectin-1, a C-type lectin receptor (CLRs), recognizes the cell wall component β-glucan. Loss of Dectin-1 results in uncontrollable *A. fumigatus* growth due to impaired neutrophil recruitment (8). Similarly, the melanin-sensing C-type lectin receptor (MelLec) was found to be protective against *A. fumigatus* during systemic infections as it can sense the unit of DHN-melanin in conidia (9). G-protein coupled receptors (GPCRs) are also important for immune responses to *A. fumigatus*. For instance, deletion of the leukotriene B4 receptor (LTB4R) in mice results in a deficiency in neutrophil and eosinophil recruitment and increased susceptibility to infection (10).

The orphan GPCR G2A, also known as GPR132, was first named due to the accumulation of cells at the G2/M cell cycle stage when the receptor is overexpressed (11). G2A is expressed on most leukocytes, including macrophages, neutrophils, and lymphocytes, and has been associated with cell recruitment, polarization, and autoimmune pathologies (12–14). Some of the known ligands for G2A include specific oxygenated short-chain fatty acids known as oxylipins. Two oxylipins, 9-hydroxyoctadecadienoic acid (9-HODE) and 13-HODE, have been shown to activate G2A (15). Both 9-HODE and 13-HODE are metabolized from linoleic acid by mammalian oxygenases (16, 17). *A. fumigatus* possesses similar oxygenases (PpoA and PpoC) (18–20) that metabolize linoleic acid into fungal specific oxylipins, 5,8-diHODE, 8-HODE,10-HODE, with structural similarity to 9-HODE and 13-HODE (**Table 1**) (19). The PpoA metabolites, 8-HODE and 5,8-diHODE, are critical signaling molecules directing fungal developmental switches (21) and all HODEs, both fungal and vertebrate, are putative ligands of specific fungal GPCRs (22, 23).

**Table 1:** Oxylipin production in *A. fumigatus* infected lung.

Oxylipin	Structure	Naïve (nmol/g lung)	D2 (nmol/g lung)	D3 (nmol/g lung)
5,8-diHODE	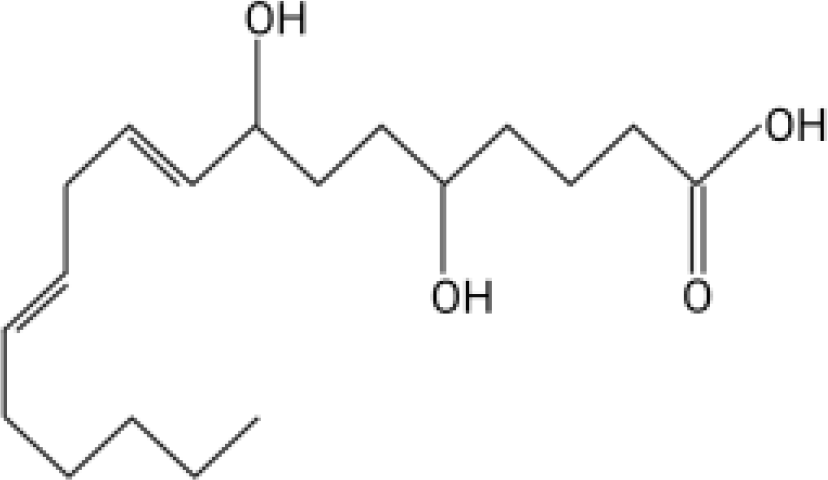	0.022 ± 0.02	5.28 ± 3.92	3.21 ± 1.47
8-HODE	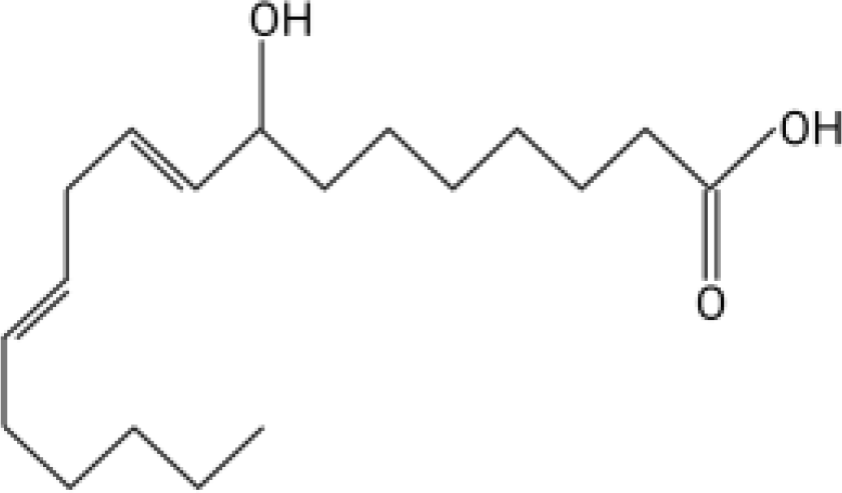	0.470 ± 0.09	127.6 ± 76.4	60.4 ± 29.6
9-HODE	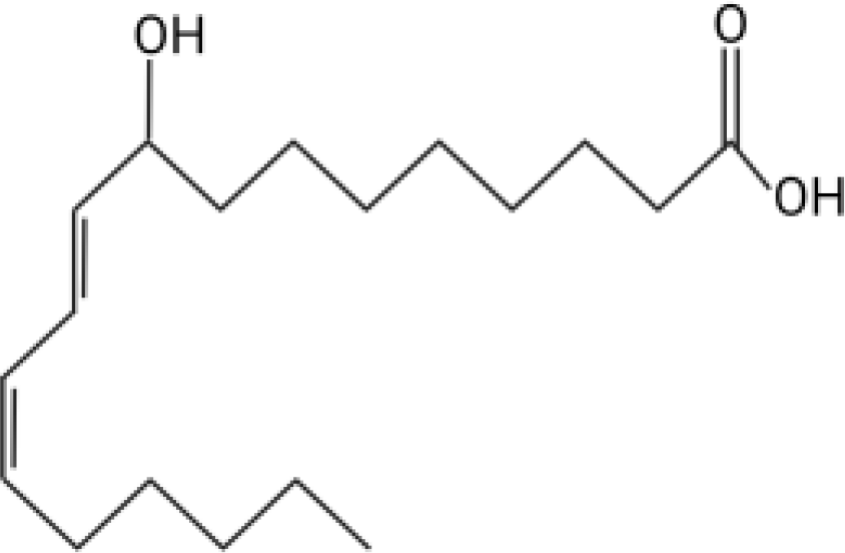	0.217 ± 0.05	1.12 ± 3.4	0.124 ± .033*
13-HODE	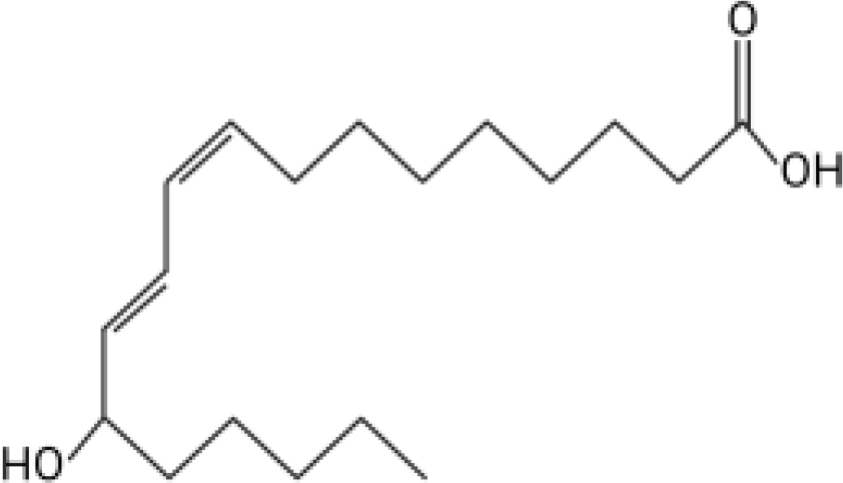	0.180 ± 0.04	0.972 ± 0.31	0.163 ± .05

*Represents statistical differences between D2 and D3 p.i.

Concentrations are listed as averages ± SEM.

Considering the relationship of fungal oxylipins with endogenous *Aspergillus* GPCR activity (22, 23), and because *ppoA* is upregulated during pulmonary infection and presumably resulting in the synthesis of fungal oxylipins (24), we hypothesized that *A. fumigatus* could modulate host immune responses through ligand interference of G2A. *In vitro* assays showed potential agonist activity of 8-HODE for G2A and partial antagonist activity of 5,8-diHODE. Survival experiments showed G2A^-/-^ mice are more resistant than G2A^+/+^ mice to the development of invasive aspergillosis. Decreased fungal virulence in G2A^-/-^ mice is correlated with increased recruitment of neutrophils and increased production and/or altered kinetics of several chemokines and cytokines shown in prior studies to be important in host defense against *A. fumigatus*. However, it remains unclear if the fungal oxylipins are important in the G2A-mediated response to *Aspergillus* due to the variation of fungal oxylipin content in infected murine lung tissue.

## Materials and methods

### Fungal oxylipin purification

To synthesize each oxylipin, 5,8-diHODE and 8-HODE, from the precursor, linoleic acid (LA), appropriate enzymes were produced by culturing *E. coli* strains (**Supplemental Table 1**). The conditions for the culturing plasmid and enzymatic reaction with LA followed previous publications (25, 26). High pressure liquid chromatography (HPLC) separations were performed on a Gilson preparative HPLC system equipped with a 332 pump and a 171 DAD detector. Ultra-high-pressure liquid chromatography–high resolution mass spectrometry (UHPLC–HRMS) data were acquired using a Thermo Scientific Q Exactive Orbitrap mass spectrometer coupled to a Vanquish UHPLC. All solvents used were of spectroscopic grade. Detailed compound isolation can be found in the supplemental materials and methods.

### GPCR agonist/antagonist assays

Purified compounds, 5,8-diHODE and 8-HODE, (**Supplemental Figure 1**), were screened against G2A at concentrations of 1, 10, and 100 μM for agonist and antagonist activity using the PathHunter β-arrestin assay (DiscoverX, Eurofins, Freemont, CA). The control ligand used for this assay was (±)9-HODE. The data were normalized to the maximal and minimal response was observed in the presence of control ligand and vehicle. For antagonist assays, the data were normalized to the maximal and minimal response observed in the presence of the (±)9-HODE at its EC80 concentration (16.7 μM). Samples were run as duplicates. Partial activity was characterized by between 20-50 percent efficacy while full activity was characterized by greater than 50 percent efficacy (**Supplemental Table 3**).

### Mice

Sex-matched G2A^+/+^ and G2A^-/-^ mice (Jackson Laboratories, Bar Harbor, ME, USA) or C57BL6/J mice (UW Madison Breeding Core, Madison, WI) between 6-14 weeks old were used in the following experiments. All experiments were performed according to the Guide of the Care and Use of Laboratory Animals of the National Institutes of Health and were approved by the University of Wisconsin-Madison Animal Care and Use Committee.

### Murine fungal infection models

*Aspergillus fumigatus* A1163 (also known as CEA10) was grown as an overlay on glucose minimal media (GMM) (18) for 3 days at 37°C. Spores were harvested in water supplemented with 0.01% Tween 80, washed with PBS three times, and enumerated using a hemocytometer to resuspend in PBS to the required concentration for the respective infection assay. For the chemotherapeutic model, mice were injected intraperitoneally (IP) four days prior to infection with cyclophosphamide (200 mg/kg). One day prior to infection, the mice are injected via IP with cyclophosphamide (200 mg/kg) and subcutaneously (SC) with triamcinolone (40 mg/kg). During immunosuppression, mice are given enrofloxacin (0.17 mg/mL) in their drinking water to prevent bacterial infection. Mice are infected at day zero with 2x10^6^ conidia/50 μL intranasally (IN) or received PBS as a control.

For the immunocompetent model, naïve mice are infected with 3x10^8^ conidia/50 μL or PBS via IN based on a published protocol (27). During all procedures, the mice were anesthetized using isoflurane. For survival assays, the animals were monitored twice daily for ten days following infection, and each treatment group, unless otherwise indicated, contained ten mice. Endpoints for the animals included those that reached a moribund state and/or those that lost greater than 20% of their original weight. For immunological assays, animals were infected as previously stated and removed at D1, D2, and D4 p.i. Experiments were repeated to ensure reproducibility.

### Oxylipin analysis

The left lobe of mice lungs were isolated from naïve (n=5) and immunosuppressed mice at days two (n=5) and three (n=4) post-infection with wild type *A*. *fumigatus* A1163 (listed as Af). The lung tissues were flash frozen and stored at -80C until lipid isolation and quantification based on previously published work (28–30). Briefly, an antioxidant cocktail solution (10 μL of 0.2 mg/mL of butylated hydroxytoluene (BHT) and EDTA in methanol and water solution) and 10 μL of a 100 nM isotope internal standard solution (including d4 6 keto PGF1a, d4 TXB2, d4 PGE2, d4 LTB4, d6 20 HETE, d11 14,15 DiHETrE, d8 12 HETE, d8 5 HETE, d4 9 HODE, and d11 11,12 EpETrE) were added to the frozen tissue and each sample was homogenized with 400 μL of ice-cold methanol and 0.1% of acetic acid and 0.1% BHT at 30 Hz for 10 min and stored at -80°C overnight. The following day, samples were centrifuged at 10,000 rpm for 10 min and supernatants were collected. The remaining pellets were washed with 100 μL of ice-cold methanol with 0.1% acetic acid and 0.1%BHT and centrifuged. The supernatants of each sample were combined and diluted with water (2 mL). The samples were loaded onto oasis HLB solid phase extraction (SPE) cartridges and the SPE protocol was used to extract the fatty acids as previously described (28). A modified approach was used to confirm that the fungal-derived oxylipins were produced by *A. fumigatus* by comparing D2 p.i. mice infected with a CEA17Δ*ku80* to a Δ*ppoABC* strain (TMN32.1) devoid of oxylipin production (31), along with naïve and A1163 controls (supplemental materials and methods).

The UPLC–MS/MS measurements were made using a Sciex 6500+ QTRAP system (Sciex, Redwood, CA, USA) hyphenated to a Waters Acquity UPLC system. The mass spectrometer was operated under the scheduled MRM mode with an electrospray ion source. All parameters were optimized using authentic standards and the quantification was carried out against the calibration curve from 0.25 to 800 nM standard calibration solutions (28–30).

### Cytokine analysis

Mice were sacrificed at D1 and D2 p.i. via CO2. Following the protocol described in Mackel et al. (32) with some minor modifications, the right lung (superior, middle, inferior, and post caval lobes) was excised from each mouse and digested with collagenase IV (1 mg/mL). RBCs were lysed and cells were enumerated and plated in a tissue culture plate at 1 x10^6^ cells/200 μl in modified IMDM. Following a 24h incubation, the samples were centrifuged, and supernatants were collected and frozen until analysis using multiplex analysis to assess inflammatory cytokines (including MCP-1, MIP-1α/β, MIP-2, KC, Eotaxin, MIG, G-CSF, and VEGF).

### Histological analysis

Using the University of Wisconsin-Madison’s Department of Surgery Histology Core, formalin-fixed, paraffin-embedded lungs were cut longitudinally across the coronal plane in 5-μm sections, mounted on glass slides, and stained with hematoxylin and eosin (H&E) for inflammation. The slides were imaged by the staff at UW Madison Translational Research Initiatives in Pathology (TRIPath) Center using the Aperio Digital Pathology Slide Scanner with the 40x brightfield imaging system. Photomicrographs were analyzed using the Aperio ImageScope software. To assess stained samples, tools in the software were used to highlight areas of inflammation. The percentage of inflammation was determined by calculating the total area of inflammation within the respective lung in comparison to the whole area of the lung (**Supplemental Figure 4**).

### Flow cytometric analysis

To prepare lung single-cell suspensions, tissue was minced and subjected to collagenase IV (Thermo Fisher) digestion with gentle agitation at 37°C for 1h. RBC lysis buffer was used to remove red blood cells following the manufacturer’s protocol. Cells were fixed with 10% NBF and washed before resuspending to 1 x10^7^ cells/mL in PBS with 1% BSA.

Cells were incubated with an Fc block and stained with Abs (listed in **Supplemental Table 2**). Flow cytometry was performed on a BD LSRII or the ThermoFisher Scientific Attune NxT flow cytometers. Data were analyzed on FlowJo 10.8.0. See **Supplemental Figure 3** for gating strategy. Briefly, CD45^+^ myeloid cells were assessed for Ly6G^+^ populations to characterize neutrophils, and Ly6G^-^ populations were characterized using CD11b and CD11c to identify monocytes, macrophages, and dendritic cell populations.

### Statistics

The logrank (Mantel-Cox) test for survival differences and the Student’s *t* test with Welch’s correction were used to assess statistical differences in the data presented in this study Analysis utilized GraphPad Prism version 9.4.1 for Windows (GraphPad Software, San Diego, California USA). Statistical differences are shown as *p* values on the respective graphs. Data is considered statistically significant if *p ≤* 0.05.

## Results

### *Aspergillus fumigatus* produces 5,8-diHODE and 8-HODE during pulmonary infection

Because PpoA has been reported to be highly expressed in murine tissue (24), we were interested to see if its products 5,8-diHODE and 8-HODE were detectable in the lungs of infected mice. We utilized a chemotherapeutic model to assess the production of these oxylipins during pulmonary infection. Both oxylipins were detectable in mice infected with *A. fumigatus* A1163. 8-HODE was the most abundant (127.6 ± 76.4 nmol/g lung) but 5,8-diHODE (5.3 ± 3.9 nmol/g lung) was also detected at D2 post-infection (**Table 1**), a time point corresponding with early innate immune responses in mice. 9-HODE and 13-HODE were also elevated at D2 p.i. compared to naïve controls, although at considerably lower levels than the fungal oxylipins (ca. 1 nmol/g lung, **Table 1**). To confirm that these oxylipins were truly derived from *A. fumigatus*, we then compared oxylipin production in mice infected with either a mutant deleted for all *ppo* enzymes (Δ*ppoABC*, TMN32.1) (31), the parental of TMN32.1 (CEA10ΔKU80, containing all 3 *ppo* genes) along with naïve and A1163 controls. 5,8-diHODE and 8-HODE were only detected in the lungs of the mice infected with wild type *A. fumigatus* (**Supplemental Figure 2**).

High variability was found in the samples from mice at D2 and D3 p.i., which was likely attributed to differences in fungal growth between mice hosts. For this study, only the left lobe was collected and fully processed for lipidomic profiling. As such, the fungal burden and progression of the disease could not be verified. There was a greater consistency amongst the mouse-derived oxylipins.

### *In vitro* assays suggest 8-HODE is an agonist and 5,8-diHODE a partial antagonist of G2A

Direct communication between the host immune system and invading microorganisms is a critical area to investigate to not only understand the disease process but also develop new therapeutics to treat infections. Considering the structural similarity of 8-HODE and 5,8-diHODE to the known human G-protein coupled receptor G2A ligands 9-HODE and 13-HODE (**Table 1**), we sought to determine if *A. fumigatus* oxylipins could interact with G2A. G2A activation was measured using the PathHunter β-Arrestin assay where G2A β-galactosidase activity results in a quantifiable chemiluminescent signal. Antagonist activity is detected through the loss of the chemiluminescent signal when a compound interferes with the activation of G2A.

**Figure 1:**
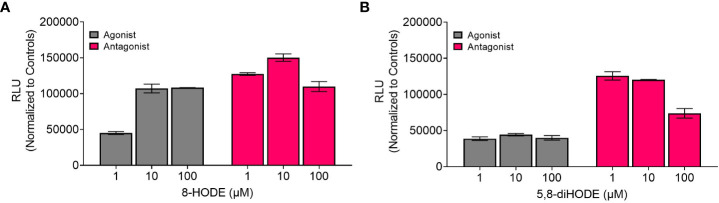
8-HODE is an agonist and 5,8-diHODE is a partial antagonist of G2A. Using the PathHunter β-arrestin assay (DiscoverX, Eurofins) to assess changes in luminescence we screened 8-HODE **(A)** and 5,8-diHODE **(B)** at 1, 10, and 100 μM for agonist (grey bars) and antagonist (pink bars) activity. Data was normalized by DiscoverX to the maximal and minimal response observed in the presence of control ligand ((±)9-HODE) and vehicle (DMSO) respectively. For antagonist assays, the data was normalized by DiscoverX to the maximal and minimal response observed in the presence of the (±)9-HODE at its EC80 concentration (16.7μM) with the vehicle. Samples were run as duplicates. Partial activity was characterized by between 20-50 percent efficacy while full activity was characterized by greater than 50 percent efficacy (**Supplemental Table 3**).

We tested three different concentrations of each *Aspergillus* oxylipin (1, 10, 100 μM) for agonist and antagonist activity on G2A based on the manufacturer’s suggestion as the EC50 for 9-HODE is at 6.23 μM for the agonist assay. At 10 μM, 8-HODE showed increased relative luminescence (RLU) (**Figure 1A**, gray bar) with an efficacy greater than 50% (**Supplemental Table 3**) suggesting that 8-HODE is an agonist of G2A. 5,8-diHODE showed no agonist activity (**Figure 1B**, gray bar) though there was a slight decrease in RLU at 100 μM indicating partial antagonism of the G2A receptor against 9-HODE at EC80 levels (16.7 μM) (**Figure 1B**, pink bar). 8-HODE did not show antagonist activity against G2A (**Figure 1A**, pink bar).

### Survival advantage for G2A^-/-^ mice infected with *A. fumigatus* is not associated with fungal clearance

**Figure 2:**
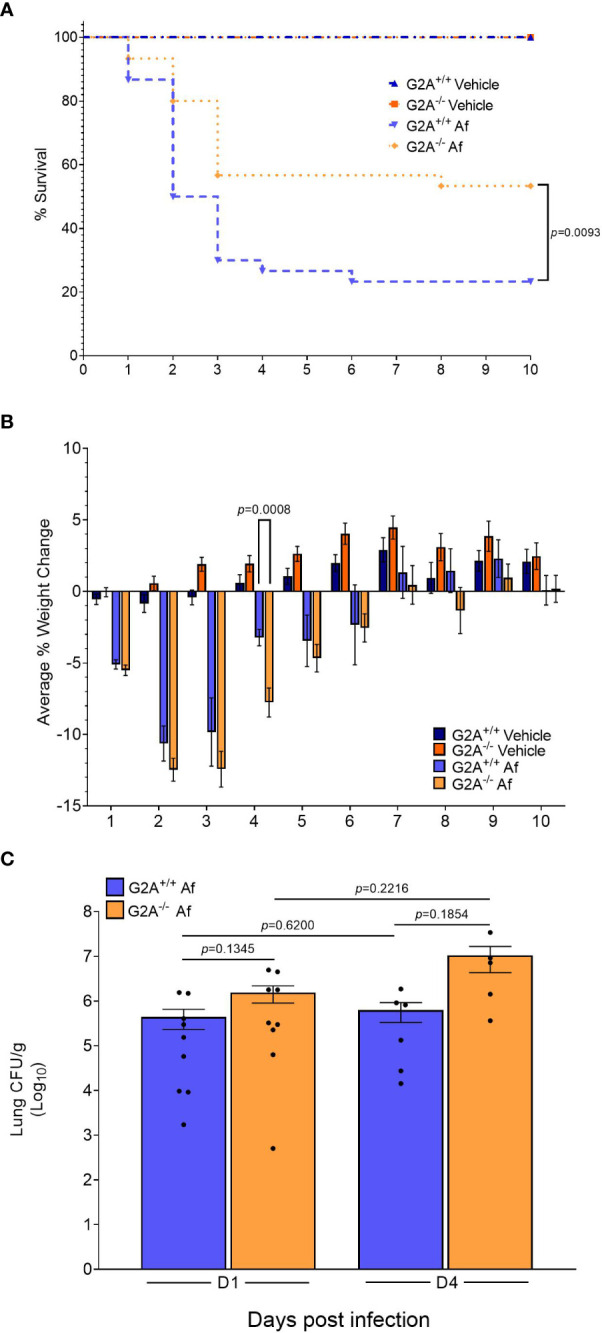
Survival advantage for immunocompetent G2A^-/-^ mice. An immunocompetent model was used to assess differences in survival **(A)** between G2A^+/+^ and G2A^-/-^ mice. Infection with *A fumigatus* is represented in the graph as Af. Pooled samples from three independent studies for G2A^+/+^ Vehicle (n=22, dark blue triangle), G2A^-/-^ Vehicle (n=22, dark orange square), G2A^+/+^ Af (n=30, blue upside-down triangle), and G2A^-/-^ Af (n=30, orange diamond) are shown as percent survival over time (days). A log-rank (Mantel-Cox) test was used to assess statistical differences. The statistical difference between G2A^+/+^ Af and G2A^-/-^ Af is shown as a *p* value. Weight changes were tracked over time for all the above mentioned groups and shown as average percent weight change ± SEM **(B)**. Statistical differences were assessed between all groups using the Student’s *t* test with Welch’s correction and samples that were statistically different between G2A^+/+^ Af and G2A^-/-^ Af is listed as a *p* value. Fungal burden **(C)** was assessed from a subset of samples at D1 and D4 post-infection via CFU/g lung tissue. The average ± SEM is shown with individual mice shown as circles within the bar graphs. Statistical differences were assessed via the Student’s *t* test using Welch’s correction and *p* values are listed to denote differences between G2A^+/+^ and G2A^-/-^ mice.

The findings that the fungal oxylipins were present in host lungs (**Table 1**) and that each oxylipin either bound or inhibited 9-HODE binding of G2A during the agonist and antagonist assays (**Figure 1**) led us to ask if G2A could impact the severity of fungal disease. We utilized an immunocompetent model of infection in G2A^+/+^ and G2A^-/-^ mice to preserve the G2A-related immunological function in a healthy host. By D10 p.i., approximately 80% of the G2A^+/+^ animals had succumbed to infection in comparison to the G2A^-/-^ animals which had a survival rate of around 50% (*p=*0.0093) (**Figure 2A**). During the infection, we noted that the G2A^-/-^ animals had an average weight loss at D4 p.i. that was significantly greater than that of the G2A^+/+^ animals and the vehicle controls, though the difference in the weight loss between the G2A^+/+^and G2A^-/-^ mice reduced following D4 p.i. (**Figure 2B**). Because the survival difference was significant, we wanted to determine if there was a difference in fungal burden. However, we found no significant differences in CFU/g of lung tissue in the G2A^+/+^ *vs* G2A^-/-^mice at D1 p.i. and D4 p.i. (**Figure 2C**).

### Increased chemokine expression in G2A null mice

**Figure 3:**
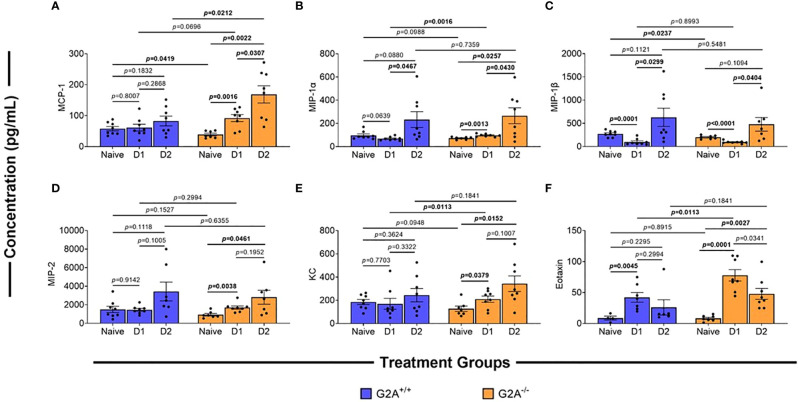
Differences in chemokines of G2A^-/-^ mice in comparison to wild-type. Cells were isolated from lungs of naïve, D1, and D2 samples post-infection of G2A^+/+^ (blue bars) and G2A^-/-^ (orange bars) mice. Cells were incubated for 24h in modified IMDM. Supernatants were collected and analyzed using multiplex analysis to assess inflammatory chemokines MCP-1 **(A)**, MIP-1α **(B)**, MIP-1β **(C)**, MIP-2**(D)**, KC **(E)**, and Eotaxin **(F)**. Concentrations (pg/mL) are listed as average protein ± SEM from two independent studies pooled (individual values, circles). Statistical differences were assessed using the Student’s *t* test with Welch’s corrections with *p* values listed on graphs.

As fungal burden did not seem to underlie the differences in virulence, we hypothesized that changes in immune responses could explain, at least in part, the increased survival of the G2A^-/-^ mice. We assessed changes in chemokine profiles of cells isolated from the lungs of mice at D1 and D2 p.i. The monocyte chemoattractant protein (MCP)-1 which is involved in the recruitment of monocytes, dendritic cells, and memory T cells to the sites of inflammation, has been found to be increased during *A. fumigatus* infection (33). MCP-1 is produced by alveolar epithelial cells through dectin-1 or CR3 receptors upon interactions with swollen *A. fumigatus* conidia (34). When we characterized this protein in the G2A^+/+^ animals there was no significant difference within the G2A^+/+^ animal treatment groups (**Figure 3A**, blue bars). In contrast, there was a significant increase in MCP-1 in G2A^-/-^ mice between naïve samples and samples from D1 (*p*=0.0016) and D2 p.i. (*p*=0.0022). There was also a significant increase in the concentration of MCP-1 between D1 and D2 p.i. in these animals (*p*=0.0307). Comparing between G2A^+/+^ and G2A^-/-^ groups, the naïve G2A^-/-^ mice have significantly less basal levels of MCP-1 (*p*=0.0419). We also saw that at D2 p.i., the G2A^-/-^ produced more MCP-1 than that of the G2A^+/+^ animals (*p*=0.0212).

In addition to MCP-1, we also assessed other innate immune cell chemokines, including macrophage inflammatory proteins (MIP)-1α/β. A previous *A. fumigatus* infection study found that there was an increase in MIP-1α/β gene expression though there was no difference in protein production (35). However, in a clinical study, it was identified that increased concentrations of MIP-1α correlated with an increased risk of developing invasive fungal disease (36). Therefore, we assessed G2A^-/-^ and G2A^+/+^ mice for MIP-1α/β concentrations but found little difference in the production of these chemokines between the genotypes with the exception that at D1 p.i. the G2A^-/-^ showed a significant increase in MIP-1α in comparison to the G2A^+/+^ mice (**Figures 3B, C**).

Like MIP-1α/β, macrophage inflammatory protein (MIP)-2 and keratinocytes-derived chemokine (KC) also are associated with *A. fumigatus* infections as they bind to the host chemokine receptor CXC chemokine receptor-2 (CXCR2) during early responses to fungal conidia. This is critical for the neutrophil-mediated response to pulmonary fungal infection in immunocompetent individuals (37). We found MIP-2 production (**Figure 3D**) in the G2A^+/+^ treatment groups remained relatively steady, while there was a significant increase in this protein for the G2A^-/-^ animals at D1 (*p*=0.0038) and D2 (*p*=0.0461) p.i. when compared to the naïve counterpart. However, when we compare these values to G2A^+/+^, there was no statistical difference. A similar trend was observed in KC levels in which there was a significant increase at D1 (*p*=0.0379) and D2 (*p*=0.0152) p.i. in G2A^-/-^ comparison to naïve (**Figure 3E**).

Finally, we assessed the production of eotaxin as it is the primary chemokine associated with the recruitment of eosinophils (38). Eosinophils are often associated with allergic fungal diseases and are important in protecting the lumen against invading parasites and fungi (39–41). We saw a significant increase of eotaxin for both G2A^+/+^(*p*=0.0045) and G2A^-/-^ (*p*=0.0001) mice at D1 p.i. when compared to naïve counterparts with a more striking increase in the G2A^-/-^ mice (*p*=0.0113). For the G2A^-/-^ mice, the eotaxin levels drop slightly at D2 p.i. in comparison to D1 p.i. (*p*=0.0341) but remained elevated compared to naïve mice (*p*=0.0027).

### G2A^-/-^ mouse neutrophils are significantly increased, while macrophages and dendritic cells are significantly reduced in response to *A. fumigatus* infection

**Figure 4:**
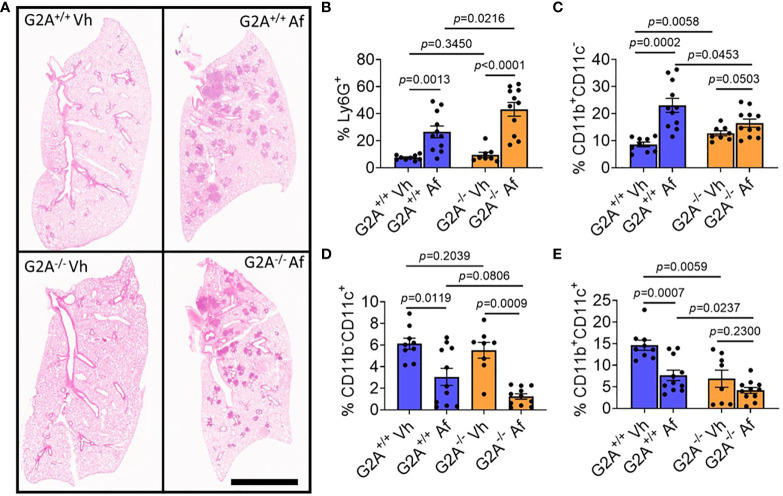
No pathological differences in inflammation, but significant differences in the myeloid population including increased neutrophilia for G2A^-/-^ mice. Representative photomicrographs of lungs from G2A^+/+^ Vh and *A. fumigatus* (Af) infected lungs (**A**, top) and G2A^-/-^ Vh and Af (**A**, bottom) are shown of H&E-stained samples. Cells isolated from lungs at D1 p.i. were assessed using flow cytometry in which populations of myeloid cells were categorized from total CD45+ cells. Percent of Ly6G^+^ cells (neutrophils, **B**), Ly6G^-^CD11b^+^CD11c^-^ cells (monocytes, macrophages, and eosinophils, **C**), Ly6G^-^CD11b^-^CD11c^+^ (alveolar macrophages and dendritic cells, **D**), and Ly6G^-^CD11b^+^CD11c^+^ (dendritic cells, **E**) from G2A^+/+^ Vh or Af (blue bars) and G2A^-/-^ Vh or Af (orange bars) are shown as average percent ± SEM with individual samples (circles) shown. Statistical differences were determined using the Student’s *t* test with Welch’s correction. *P* values are listed within the graphs comparing groups.

Overall pathological changes to lung tissue were assessed by staining with H&E for inflammation at D1 p.i. Both infected G2A^+/+^ and G2A^-/-^ samples showed similar inflammation patterns (**Figure 4A**) which were quantified and showed no statistical differences (**Supplemental Figure 4E**). Although there was no difference in visual inflammation, we thought it possible that there could be a difference in the myeloid cell populations present in the lung. Myeloid cells are essential in the host immune response against *A. fumigatus.* Neutrophils, which are Ly6G^+^, play an important role in clearing hyphal growth through degranulation and NET formation (42, 43). Infected G2A^-/-^ mice had significantly more neutrophils than infected G2A^+/+^ mice (*p*=0.0216) and both mice showed increased neutrophilia when compared to vehicle controls (**Figure 4B**).

Other myeloid cells including macrophages and monocytes phagocytose and produce cytokines to stimulate adequate immune responses against the invading fungus. In other models looking at G2A^-/-^ mice, macrophage migration to the site of inflammation was reduced due to a change in macrophage polarization (13). We assessed three different Ly6G^-^ populations for expression of CD11b and CD11c. CD11b^+^CD11c^-^ cells (**Figure 4C**), which include macrophages, monocytes, and eosinophils, were elevated in G2A^+/+^ infected mice compared to the infected G2A^-/-^ mice (*p*=0.0453). This population of cells was also elevated in G2A^-/-^ mice compared to vehicle control (*p*=0.0503). Alveolar macrophages were also assessed (CD11b^-^CD11c^+^, **Figure 4D**) and populations were statistically suppressed in both G2A^+/+^ and G2A^-/-^ mice compared to control (*p*=0.0119 and *p*=0.0009 respectively) but not between infected G2A^+/+^ and G2A^-/-^ mice (*p*=0.0806).

Finally, when we assessed the CD11b^+^CD11c^+^ population of dendritic cells (DCs), we saw that vehicle G2A^+/+^ mice had significantly more DCs than the vehicle G2A^-/-^ mice (*p*=0.0059). The DC population declined in infected G2A^+/+^ mice (*p*=0.0007) but not in infected G2A^-/-^ mice (*p*=0.0237) (**Figure 4E**).

### Cell differentiation and angiogenesis proteins increased significantly in G2A^-/-^ mice

**Figure 5:**
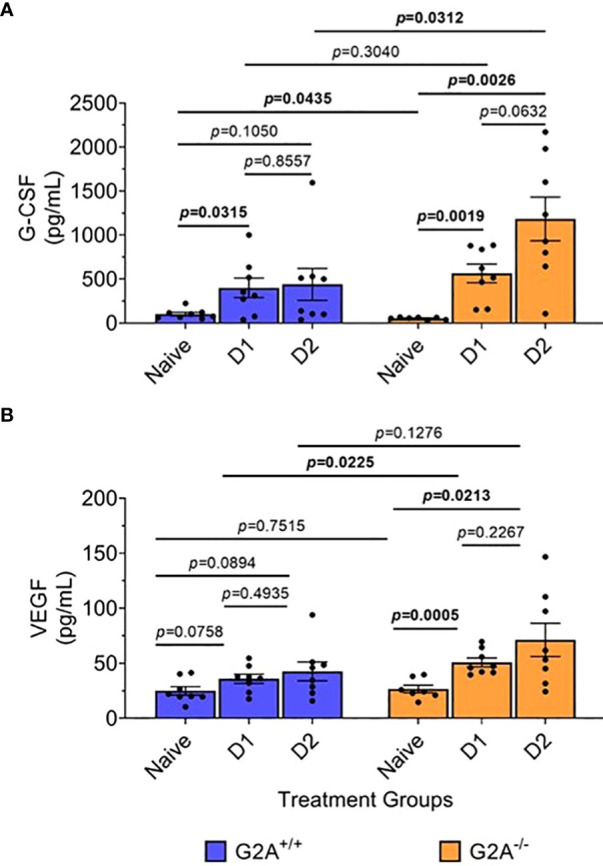
Increased G-CSF and VEGF in infected G2A^-/-^ mice. Cells were isolated from lungs of naïve, D1, and D2 samples post-infection of G2A^+/+^ (blue bars) and G2A^-/-^ (orange bars) mice. Cells were incubated for 24h in modified IMDM. Supernatants were collected and analyzed using multiplex analysis to assess G-CSF **(A)** and VEGF **(B)**. Concentrations (pg/mL) are listed as average protein ± SEM from two independent studies pooled (individual values, circles). Statistical differences were assessed using the Student’s *t* test with Welch’s corrections with *p* values listed on graphs.

The changes in some chemokines’ trends (**Figure 3**) and increased proportion of neutrophils in infected G2A^-/-^ mice (**Figure 4B**) suggested a prominent role of neutrophils in enhancing the survival of G2A^-/-^ mice. Thus, we asked if granulocyte cell stimulating factor (G-CSF) levels could be altered in infected G2A^-/-^ mice in comparison to G2A^+/+^ as G-CSF is important for neutrophil survival, proliferation, and differentiation (44, 45) and was recently shown to be involved in the suppression of *A. fumigatus* germination (45). It is also involved in damaging hyphae of *A. fumigatus* by enhancing the neutrophil oxidative burst (46). As seen in **Figure 5A**, G2A^+/+^ and the G2A^-/-^ mice showed increases in the production of G-CSF at D1 p.i. However, at D2 p.i., the concentration of G-CSF remained relatively steady in the G2A^+/+^ mice with no differences between the naïve and the D1 p.i. samples. In contrast, the G2A^-/-^ mice showed a steady increase in G-CSF over time, with significant differences at D2 p.i. to the naïve mice (*p*=0.0026). Further, at D2 p.i., the G2A^-/-^ mice had a greater concentration of G-CSF being produced in comparison to the G2A^+/+^ mice (*p*=0.0312) (**Figure 5A**). This result, together with the finding that G2A^-/-^ showed an increased neutrophil population, suggested that increased neutrophil recruitment may contribute to the more robust survival rate of infected G2A^-/-^ mice.

Finally, we asked if vascular endothelial growth factor (VEGF) levels could provide some clue to the survival advantage of infected G2A^-/-^ mice. *A. fumigatus* produces secondary metabolites, such as fumagillin (47, 48) and gliotoxin (49), that have been identified to inhibit angiogenesis during pulmonary infection. Monotherapy of VEGF reverses antiangiogenic activity by *A. fumigatus* (50). Upon examination of G2A^+/+^ mice, we found no significant differences in the G2A^+/+^ mice at D1 and D2 p.i. when compared with naïve (*p*=0.0758; *p*=0.0894 respectfully) and with each other (*p=*0.4935). However, in G2A^-/-^ mice, there was an increase in VEGF at D1 (*p*=0.0005) and D2 (*p*=0.0213) in comparison to naïve though there was no significant difference between D1 and D2 p.i. (*p*=0.2267). If we compare G2A^+/+^ and G2A^-/-^ mice, at D1 p.i. we saw a significant increase in VEGF in G2A^-/-^ mice (*p*=0.0225) but not at D2 p.i. (*p*=0.1276), likely due to the highly variable response at D2 p.i. for the G2A^-/-^ mice (**Figure 5B**).

## Discussion

The propensity of certain individuals to develop severe invasive aspergillosis is not always clear. Although a general weakness in the immune status is associated with this disease, specific host loci linked with IPA are just now being understood. For example, polymorphisms in *ARNT2* and *CX3CR1* genes, encoding a transcription factor associated with the aryl hydrocarbon receptor complex and a GPCR respectively, have been associated with more severe disease in a subset of high-risk hematological patients (51). Other genetic polymorphisms that are associated with susceptibility to invasive aspergillosis include *DECTIN1* Y238X, which results in diminished Dectin-1 receptor activity (52), and hyper responses to Danger Associated Molecular Patterns (DAMPs) associated with genetic variations in *S100B/RAGE* (53). Likewise, there were three markers in the chemokine (C-X-C motif) ligand 10 (CXCL10) that were associated with an increased risk for developing IPA as the polymorphisms determine CXCL10 production during infection (54). Notably, these mutations and several others (55) are all associated with susceptibility to IPA as most candidate gene studies are designed to find disease-prone loci. Our hypothesis that G2A could play a role in IPA was open regarding its potential as a susceptibility or resistance locus until gaining experimental evidence which in this study suggests that G2A provides a deleterious role in IPA development.

Research on G2A’s role in disease has been predominantly focused on noncommunicable diseases including nerve injury (56, 57), cardiovascular disease (58), and tumor development (59), except for two studies of commensal bacteria (60, 61). One research group found G2A gene expression to be higher in acne lesions caused by *Propionibacterium acnes* development and concluded that the receptor could play a role in quelling inflammatory cytokine response to the bacterium (60). In a second study looking for commensal bacteria effector genes, a *Bacteroides* spp. gene was found to encode for N-acyl-3-hydroxypalmitoyl-glycine, which the authors found to be a G2A agonist (61). This led the authors to speculate that N-acyl-3-hydroxypalmitoyl-glycine and other microbial GPCR ligands not only mediate host-microbe interactions but provide means towards future therapies of human infectious disease.

Our study complements the above finding. Using G2A-deficient mice, we found that there is a survival advantage when G2A is absent that is associated with neutrophilia and increased VEGF and G-CSF. We see increases in MCP-1 and MIP-1α (**Figure 4**), which are chemoattractants for neutrophils. Neutrophils are essential for host defense against *A. fumigatus* through degranulation and production of NETs that inhibit hyphal growth by sequestering zinc, though there are conflicting reports regarding the overall efficacy of NETs during infection (42, 43, 62, 63). Additionally, neutrophils can phagocytose fungal spores, though are not as effective as macrophages (64). G-CSF signals regulate neutrophil release from the bone marrow and contribute to neutrophil viability and resistance to *A. fumigatus* infection by suppressing neutrophil apoptosis (65). There have been several studies that have examined the impact of G-CSF on neutrophil function and host survival during infection. In mouse infections, G-CSF administration has been overall effective in protecting non-neutropenic mice from invasive candidiasis and is effective for mice with less severe neutropenia infected with *A. fumigatus* (46). Recent work has shown that G-CSF enhances the suppression of *A. fumigatus* germination (45). Our work shows that G2A may dampen neutrophilic recruitment during initial inflammation with *A. fumigatus.* Enhanced neutrophil release from the bone marrow of G2A deficient mice is shown in this study to be a survival advantage against infection.

This enhanced neutrophilia is likely contributing to the increase in VEGF production by G2A^-/-^ mice when compared with naïve and G2A^+/+^ mice during early inflammation. Neutrophils will produce VEGF during inflammation to promote vascular permeability and the formation of new vasculature (66). Fungal secondary metabolites, including fumagillin (47, 48) and gliotoxin (49), inhibit angiogenesis resulting in more severe outcomes of IPA. Inhibition of VEGF by monoclonal antibody treatment results in increased susceptibility to fungal infection in a clinical case of aspergillosis (67). In work by Ben-Ami et al., it was found that when neutropenic mice were treated with VEGF during infection they saw a prolonged survival in their mice, like what we see in our animals (50). With enhanced VEGF and G-CSF, as well as increased neutrophil recruitment when G2A is absent, we conclude that G2A plays a role in the initial responses to *A. fumigatus* that led to increased risk to succumb to the infection.

This work was initiated by the possibility that G2A could be involved in the progression of IPA due to the structural similarity of *A. fumigatus* oxylipins to the known G2A ligands, 9-HODE and 13-HODE (19). Although we found both 8-HODE and 5,8-diHODE present in *A. fumigatus* wild type infected lung tissue (**Table 1**) and the β-arrestin bioassay indicated that 8-HODE possessed agonist activity and 5,8-diHODE partial antagonist activity *in vitro* (**Figure 1**), the presence of the fungal oxylipins was not consistent in lung tissue and thus our data does not support or refute a definitive role for fungal oxylipins in G2A mediated responses to *A. fumigatus* infections. Future studies including whole lung metabolomics, infections with appropriate fungal mutants (both deletion and overexpression oxylipin mutants) coupled with further studies of neutrophil release and trafficking in G2A^-/-^ mice compared to G2A^+/+^ mice should bring greater clarity to the role of G2A in IPA.

## Data availability statement

The original contributions presented in the study are included in the article/**Supplementary Material**. Further inquiries can be directed to the corresponding author.

## Ethics statement

The animal study was reviewed and approved by University of Wisconsin-Madison Animal Care and Use Committee.

## Author contributions

BS, MN, and NK contributed to the conception of the study BS, MN, JY, BH, CS, and NK contributed to the study design. BS did all animal studies, data analysis, and statistics. SP purified the oxylipins that were used in assays. DC assisted in animal studies. MJ ran the multiplex analysis. BS wrote the first draft of the manuscript. SP and JY wrote sections of the manuscript. All authors contributed to the article and approved the submitted version.
