# Supplementary material for: Loss of the mammalian G-protein coupled receptor, G2A, modulates severity of invasive pulmonary aspergillosis
Source: Front Immunol. 2023 Jun 26;14:1173544. doi: 10.3389/fimmu.2023.1173544 (PMC10331294; doi:10.3389/fimmu.2023.1173544)
Supplement: Supplementary file 1 [file DataSheet_1.pdf]

Supplemental Material and Methods:

Compounds Isolation. To synthesize each oxylipin from the precursor, linoleic acid (LA), appropriate enzymes were produced by culturing *Escherichia coli* strains (Supplemental table 1). For protein expression, each *E. coli* strain was cultivated (37°C, 200 rpm) in a 2 L flask containing 500 mL of Luria–Bertani (LB) medium and appropriate antibiotics (either 20 μg/mL of ampicillin or 50 μg/mL of kanamycin). When the optical density (OD_{600}) of the culture reached 0.6, isopropyl-β-D-thiogalactopyranoside (IPTG)(0.1 mM final concentration) was added to induce enzyme expression. The culture was further incubated at 16°C with shaking (150 rpm) for 16 h. The enzymatic reaction with LA were performed following previously published protocols (1,2).

5,8-diHODE was isolated from the bacterial extract by preparative reversed-phase HPLC (Xbridge PREP C18 OBD column, 5 μm, 19 X 250 mm) using a gradient solvent system (30% CH_3CN-H_2O to 45% CH_3CN-H_2O over 20 min, UV detection at 210 nm, and flow rate = 16 mL/min) and isolated (t_R = 15.8 min). The 8-HODwas isolated from the extract by the same preparative reversed-phase HPLC (Xbridge PREP C18 OBD column, 5 μm, 19 X 250 mm) using an isocratic solvent system (60% CH_3CN-H_2O over 20 min, UV detection at 210 nm, and flow rate = 16 mL/min) to yield 8-HODE (t_R = 12.3 min) and 10-HODE (t_R = 14.7 min). The purified oxylipins were isolated in the following amounts: 5.1 and 4.2 mg of 5,8-diHODE and 8-HODErespectively.

To verify compound purity, UHPLC–HRMS and UHPLC–HRMS/MS were performed on a Thermo Scientific Vanquish UHPLC system connected to a Thermo Scientific Q Exactive Hybrid Quadrupole-Orbitrap mass spectrometer operated in negative ionization mode. A Waters XBridge BEH-C18 column (2.1 × 100 mm, 1.7 μm) was used with acetonitrile (0.1% formic

acid) and water (0.1% formic acid) as solvents with a flow rate of 0.2 mL/min. The screening gradient method for the extracts started at 55% mobile phase with a linear increase over 17 min to a 98% mobile phase. Ten microliters of each isolate were injected into the system for analysis. 5,8-diHODE was detected at t_R = 3.85 min with $[M-H]^-$ = 311.2232 and its known fragment, 173.0811 (1). 8-HODE was detected at t_R = 8.67 min with $[M-H]^-$ = 295.2284 and its known fragment, 157.0860 (2).

Metabolite extraction from the mouse lungs

Extraction of oxylipins from mouse lungs to verify fungal production of 5,8-diHODE and 8-HODE was accomplished by following the protocol from within the manuscript with some modifications. The right sides of the lungs were harvested from vehicle (naïve) controls and animals infected with A1163 (also known as CEA10), CEA17Δ*ku80,* or TMN32.10 (Δ*ppoABC*)(3) and weighed (between 100-200 mg). The tissue was flash-frozen in liquid nitrogen and stored at -80 °C until extraction. The frozen organs were lyophilized for 2 hr and crushed until they became a powder form. Ten µL of antioxidant solution (0.2 mg/mL BHT and EDTA) and 400 µL of ice-cold methanol with 0.1% acetic acid and 0.1% BHT was added to each sample and sonicated at room temperature for 1 hr (4). The samples were centrifuged at 10,000 rpm for 10 min and supernatants were collected. Pellets were washed with 100 µL ice cold methanol with 0.1% acetic acid and 0.1% BHT and centrifuged again. Supernatants were combined and diluted with 2 mL H_2O and analyzed via UHPLC-HRMS/MS using the following parameters.

Briefly, UHPLC–HRMS data were acquired using a Thermo Scientific Q Exactive Orbitrap mass spectrometer coupled to a Vanquish UHPLC operated in both positive and negative ionization modes. All solvents used were of spectroscopic grade. Each sample was filtered with a 0.2 mm syringe filter. A Waters XBridge BEH-C18 column (2.1 × 100 mm, 1.7

μm) was used with acetonitrile (0.1% formic acid) and water (0.1% formic acid) as solvents at a flow rate of 0.2 mL/min. The screening gradient method for the samples is as follows: starting at a 55% organic hold for 1 min, followed by a linear increase to 98% organic over 18 min, holding at 98% organic for 2 min, for a total of 21 min. A quantity of 10 μL of each sample was injected into the system for the analysis. 10 – 100 ppm of 5,8-diHODE and 8-HODE were used as standards.

Supplemental Data:

Supplemental Table 1. Plasmids Used for Compound Isolation.

**Bacterial Strain**	**Plasmid name**	**Marker**	**Gene construct in plasmid**	**Oxylipin product**
T7 Express Competent *E. coli*	pWW16.2	Kanamycin	5,8-LDS/*A.nidulans* PpoA, Kan^R	5,8-diHODE
T7 Express Competent *E. coli*	pWW17.2	Ampicillin	5,8-LDS double point mutation in PpoA, Amp^R	8-HODE

Supplemental Table 2. Antibodies for Flow Cytometry.

**Target**	**Fluorophore**	**Source**	**Catalog #**
CD16/CD32	None	Fisher Scientific	50-124-40
CD45	BV650	BioLegend	103151
Ly6G	AF700	BioLegend	127622
CD11c	FITC	Thermo Scientific	11-0114-82
CD11b	AF750	Tonbo Biosciences	25-0112-U100

*The listed antibodies were used based on manufacturer suggestions.

Supplemental Table 3. GPCR G2A Agonist and Antagonist Results with %Efficacy

**Assay Target**	**Oxylipin**	**Oxylipin conc. (µM)**	**Average RLU**	**Stdev**	**%Efficacy**
GPR132(G2A) Agonist	5,8-diHODE	1	38740	2460.7	4.2
		10	44240	1640.5	9.8
		100	39900	3083	5.4
	8-HODE	1	45080	2036.5	10.7
		10	107160	6109.4	**74.8**
		100	108240	169.7	**75.9**
GPR132 (G2A) Antagonist	5,8-diHODE	1	125620	5741.7	2.7
		10	120200	452.6	8.3
		100	73840	6675.1	**55.7**
	8-HODE	1	127360	1866.8	0.9
		10	150180	5232.6	-22.4
		100	109840	6957.9	18.9

Supplemental Figure 1. 5,8-diHODE and 8-HODE Purity.

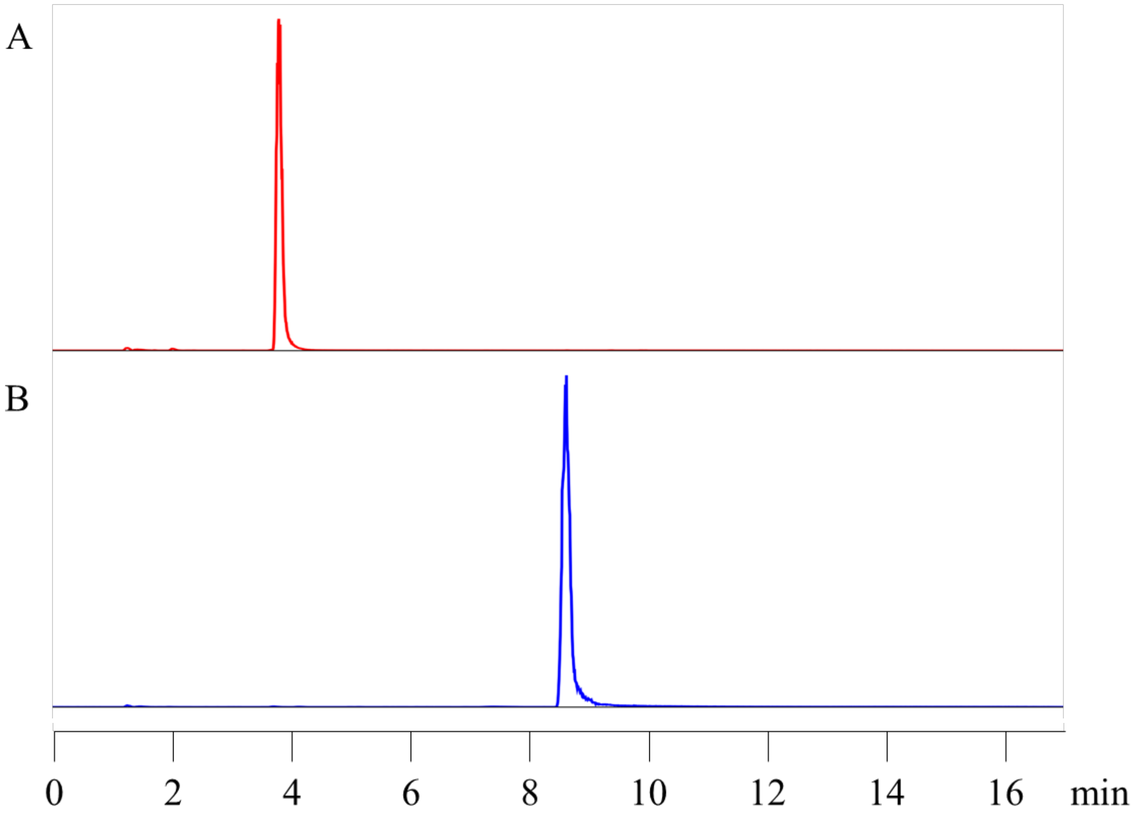

Supplemental Figure 1. Purity of the isolated each oxylipin was checked through UHPLC–HRMS/MS analysis. (A) 5,8-diHODE (t_R = 3.85 min, $[M-H]^-$ = 311.2232 with the fragment 173.0811) (B) 8-HODE (t_R = 8.67 min, $[M-H]^-$ = 295.2284 with the fragment, 157.0860).

Supplemental Figure 2. 5,8-diHODE and 8-HODE are produced by *A. fumigatus* during infection

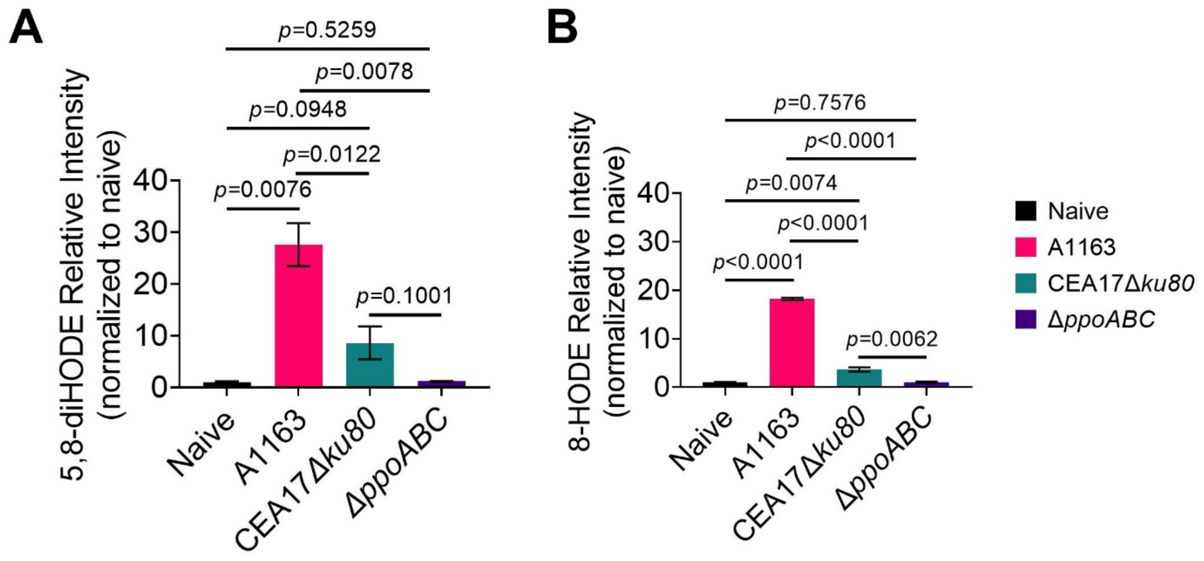

Supplemental Figure 2. 5,8-diHODE and 8-HODE are produced by *A. fumigatus* during infection. Mouse right lungs were collected and flash-frozen in liquid nitrogen on D2 p.i. until oxylipin extraction based on the method described in the supplemental materials and methods. Samples include naïve (black, n=3), A1163 (pink, n=4), CEA17Δ*ku80* (teal, n=4), and Δ*ppoABC* (TMN32.1, blue, n=4). 5,8-diHODE (A), 8-HODE (B), and 10-HODE (not shown) were analyzed. 10-HODE was not included due to difficulty detecting the oxylipin in the samples. Samples are shown as the relative intensity normalized to naïve ± SEM. Statistical analysis consisted of a Student's *t* test with Welch's correction and *p* values are listed for all comparisons.

Supplemental Figure 3. Gating Strategy for Flow Cytometry.

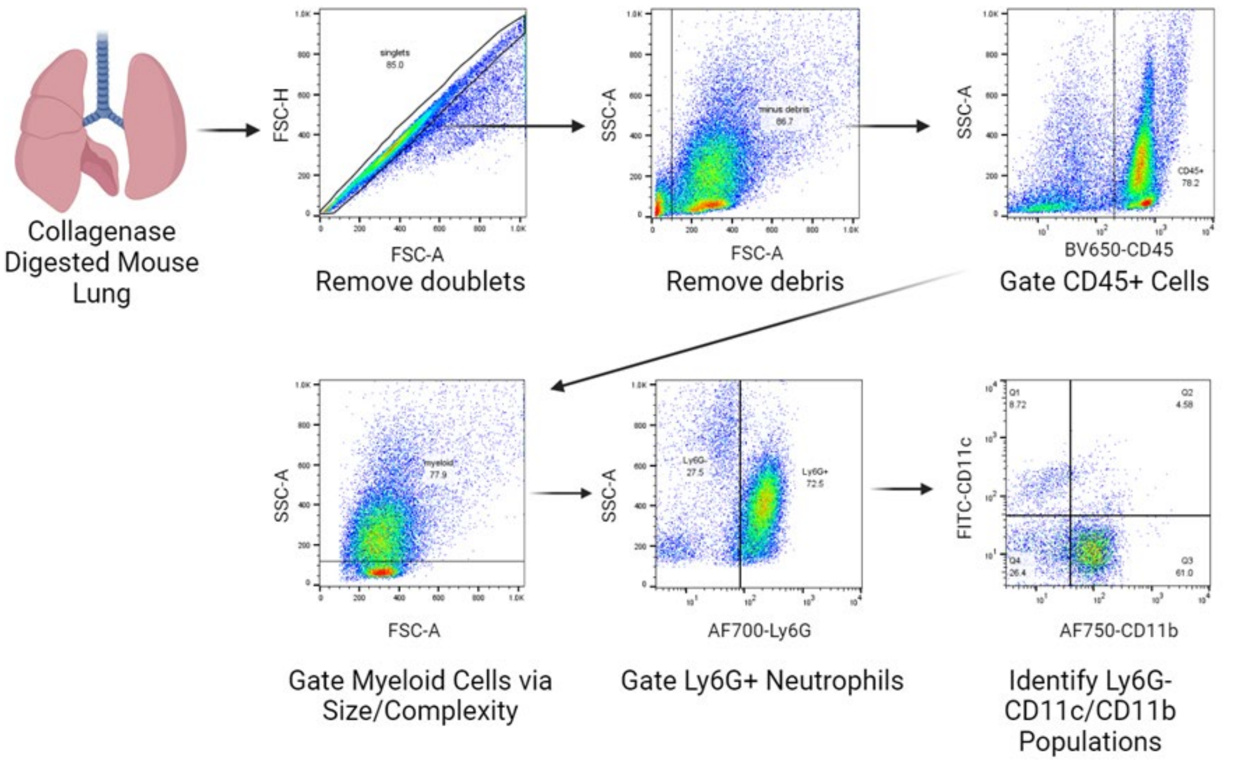

Supplemental Figure 3. Gating strategy for flow cytometric analysis. Mouse right lungs were collagenase digested and RBCs were lysed using RBC lysis buffer. Controls including cells only, single-stained populations, and populations containing all but one stain were used to discriminate the correct cell population. Doublets were removed using FSC-H and FSC-A and debris was removed consistently across all samples. CD45+ cells were gated based on complexity to isolate the myeloid population. This was followed by gating the Ly6G+ cells for neutrophils and the Ly6G- population was assessed for changes in CD11c/CD11b populations. We collected 50,000 events/lung and the average percent population is shown in Figure 4.

Supplemental Figure 4. Histopathological Analysis of Lung Inflammation

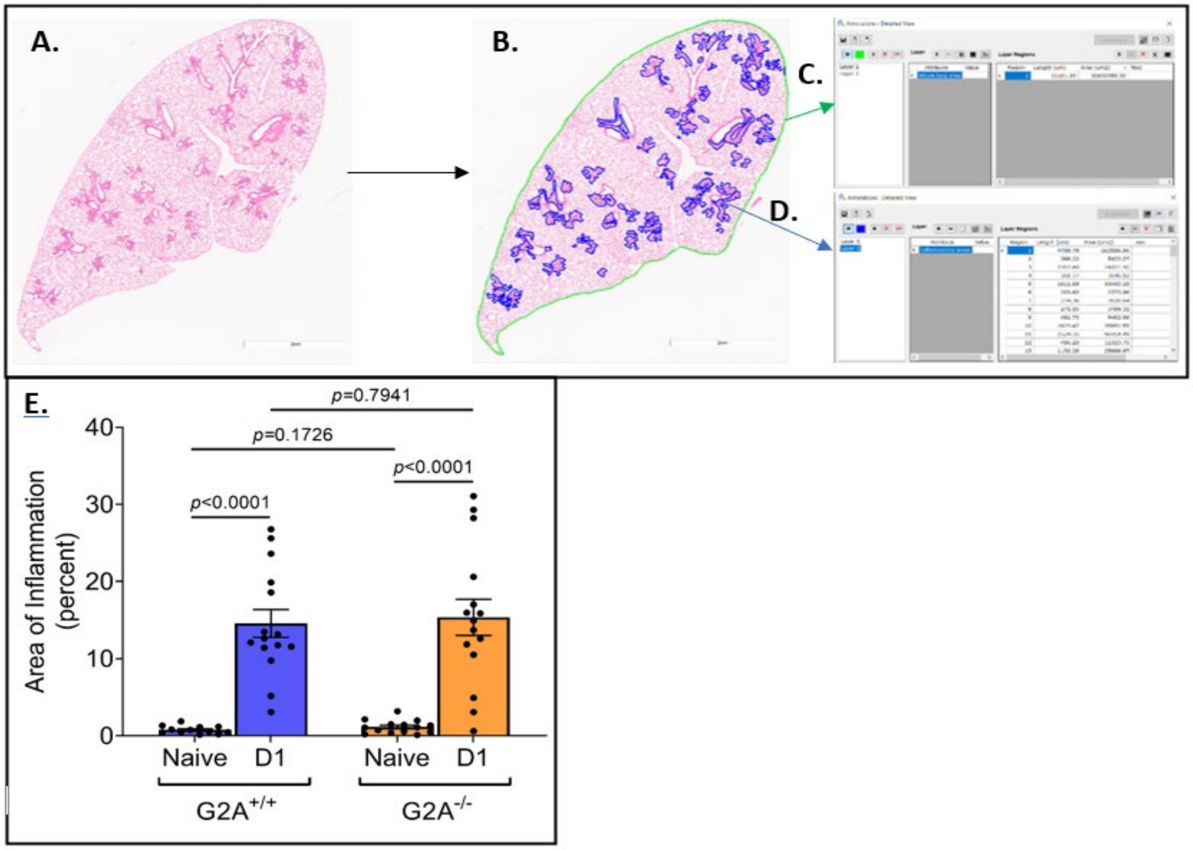

Supplemental Figure 4. Histopathological analysis of inflamed lung tissue. Left lungs were fixed and embedded with paraffin. Tissue sections were cut and stained with hematoxylin and eosin for inflammation and images were taken of the lungs using the Aperio microscope with magnification up to 40x (A.). Using the annotation software (B.) in Aperio ImageScope (12.4.3.5008), we found the total area of the lung (C.) and highlighted regions of inflammation (D.). Area of inflammation (percent) was determined by taking the average inflammation of the lung divided by the total area (E.). Individual mice are shown as single points on the respective graph and the average ± SEM is shown. Statistical differences were assessed using the Student's *t* test with Welch's correction and *p* values are listed on the graph.

Supplemental References:

1. Seo MJ, Shin KC, Oh DK. Production of 5,8-dihydroxy-9,12(Z,Z)-octadecadienoic acid from linoleic acid by whole recombinant Escherichia coli cells expressing diol synthase from Aspergillus nidulans. Appl Microbiol Biotechnol. 2014 Sep;98(17):7447–56.

2. Jeong YJ, Seo MJ, Shin KC, Oh DK. Production of 8-hydroxy-9,12(Z,Z)-octadecadienoic acid from linoleic acid by recombinant cells expressing H1004A-C1006S variant of Aspergillus nidulans diol synthase. J Mol Catal B Enzym. 2015 May 1;115:35–42.

3. Almaliki HS, Niu M, Keller NP, Yin G, Bennett JW. Mutational Analysis of Aspergillus fumigatus Volatile Oxylipins in a Drosophila Eclosion Assay. J Fungi. 2023 Apr;9(4):402.

4. Yang J, Schmelzer K, Georgi K, Hammock BD. Quantitative profiling method for oxylipin metabolome by liquid chromatography electrospray ionization tandem mass spectrometry. Anal Chem. 2009 Oct 1;81(19):8085–93.
